# Rescaling of Point Charges as a Way to Improve the Simple-to-Use Electrostatic Embedding Scheme Developed to Explore Enzyme Activity with QM-Oriented Software

**DOI:** 10.1021/acs.jcim.5c01235

**Published:** 2025-08-07

**Authors:** Andrzej J. Kałka, Aleš Novotný, Jernej Stare

**Affiliations:** † Theory Department, National Institute of Chemistry, Hajdrihova 19, 1000 Ljubljana, Slovenia; ‡ Faculty of Chemistry, 37799 Jagiellonian University, Gronostajowa 2, 30-387 Cracow, Poland

## Abstract

Computer-aided exploration of enzymatic reactions, which still leaves many important questions open, calls for robust and accurate techniques of molecular modeling. One of the most intriguing issues related to enzymatic reactions is the role of electrostatic interactions established between the reacting moiety and its enzymatic environment. In order to evaluate these interactions, we previously devised a QM/MM scheme based on electrostatic embedding of the reaction kernel, treated by quantum chemistry, into the enzymatic surroundings represented by point charges [A. Prah et al., *ACS Catal*. **2019**, 9, 1231.]. The method features remarkable simplicity and reliably predicts the effect of electrostatics on enzyme catalysis. Yet, this simplified approach has pitfalls; in particular, it tends to overestimate the attracting force between the electrons and the surrounding point charges—an effect named electron spill-out—impairing the accuracy of evaluated electrostatic interactions. Herein, by using statistical methods together with reference quantum calculations, we critically assess the impact of this pitfall and propose a very simple but effective correction based on attenuation of point charges near the QM–MM boundary depending on their distance from the quantum subsystem. We demonstrate that the proposed correction can significantly improve the accuracy of computed energies of electrostatic interactions between the reaction kernel and its enzyme surroundings, thereby representing an important methodological advance of our electrostatic embedding approach. Noteworthily, the optimal attenuation scheme can vary among the considered systems—in particular, it is sensitive to the net charge of the reaction kernel—suggesting the scheme be tuned individually for each considered enzymatic reaction following the presented workflow.

## Graphical abstract

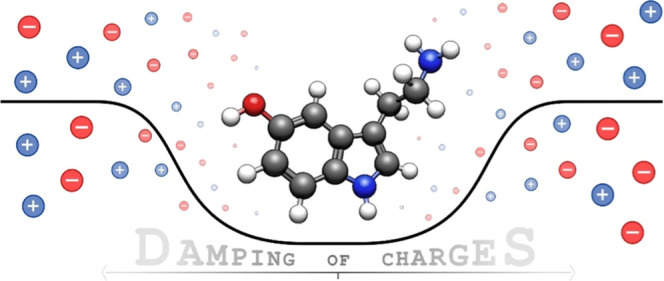

## 1 Introduction

Enzymes are biological catalysts involved in most chemical reactions occurring in life processes. Due to evolutionary pressure, enzymes evolved to be the most efficient under relatively mild conditions, increasing reaction rates to levels that make life sustainable. To avoid undesired side reactions, metabolic inefficiencies, and wasting of cellular resources, enzymes evolved to catalyze specific reactions, accepting one or only a small group of related molecules as substrates. Although the specificity and efficiency of enzymes have been the focus of intensive research for decades, the driving force behind their catalytic power still remains a hot topic of scientific debate. The predominant contribution of enzyme catalysis is currently hypothesized to arise either from dynamical effects,^1–4^ entropic effects,^5–7^ or preorganized electrostatics.^3,4,8,9^ In a nutshell, the first hypothesis claims the enzyme’s catalytic power to originate from the complex plethora of its motions, effectively triggering and shoving the reaction to occur. The second and the third theory, on the other hand, associate the activity of enzymes with lowering of the activation barrier, achieved, respectively, by mechanically restraining the substrates in their reactant state (R; reduction of the entropic penalty) or by stabilizing them in the transition state (TS; reduction of the energetic barrier) via preferential electrostatic interactions.

As regards exploring of the very nature of enzymatic catalysis, an indispensable tool for doing so is provided by computational methods, as they offer insight into very short time scales at atomistic resolution, at the same time enabling both physical and nonphysical manipulations of the system. Enzymatic reactions, especially reaction mechanisms, are commonly investigated by quantum mechanical (QM) methods as they provide accurate results and do not rely on empirical parameterization. However, the computational cost steeply increases with system size, limiting their use to systems consisting of hundreds of atoms at most.^10^ For that reason, studies using QM methods often exclude the enzymatic environment and focus solely on the reaction moiety (herein also named the reaction kernel, RK). On the other hand, molecular mechanics (MM) methods based on classical mechanics calculus are much less suitable for investigating enzymatic reactions due to their inability to explicitly treat the electronic structure and only limited ability of MM force fields to describe breaking and forming of chemical bonds. On the contrary, MM methods are excellent for explorations of conformational space of systems consisting even of millions of atoms.^11^ Finally, hybrid QM/MM methods make it possible to include the enzymatic environment using the MM method, while treating the RK with QM methods.^12^ Still, one should note that for QM/MM, the amount of required computational resources is mainly driven by the more expensive component, and in almost all cases, this is the QM part. Also, the QM part is most often (but not exclusively) based on the quantization of the electronic structure by employing standard quantum chemistry methods such as DFT.

The most common way of embedding the QM part of interest into its MM surroundings is by considering their mutual interactions in both ways.^13–15^ Specifically, the electronic structure of the QM part is effectively polarized by the surrounding MM charges, and additionally, van der Waals forces between both entities take effect. The MM part is subject to electrostatic interaction both with the electronic charge distribution and with the positively charged nuclei within the QM part. In addition, while the MM charges usually remain insensitive to electrostatic interactions with the QM part and within the MM part itself, in the case of polarizable force fields, they can also be affected by electrostatic interactions. “Traditional” QM/MM schemes include all the relevant interactions between the constituting parts and therefore fully support common simulation protocols such as energy minimization or molecular dynamics (MD) simulation and are available in a number of program packages.

Focusing on electrostatics as a possible source of enzyme catalysis, in our research group, we have developed an alternative simplified variant of the QM/MM approach, based solely on an electrostatic embedding scheme.^16^ In the cited algorithm, the enzymatic environment is represented by MM-derived point charges, which polarize the electron density of the central (reacting) moiety (treated by QM) via Coulomb interactions (Figure 1). Apart from that, no other interactions between the QM and MM part are assumed. Thus, the proposed scheme facilitates a straightforward evaluation of electrostatic interactions established between the constituents of the system. Furthermore, because of the complete absence of other nonbonding interactions, the methodology is inexpensive and trivially available in a number of quantum chemistry program packages, requiring basically no other input than QM atomic coordinates together with coordinates and values of MM point charges. Among programs supporting inclusion of point charges in QM calculations, Gaussian’s^17^ rich set of available options not only facilitates support for electrostatic embedding of the QM-treated region but also makes the prospect of this type of computation very compelling.

**Figure 1:**
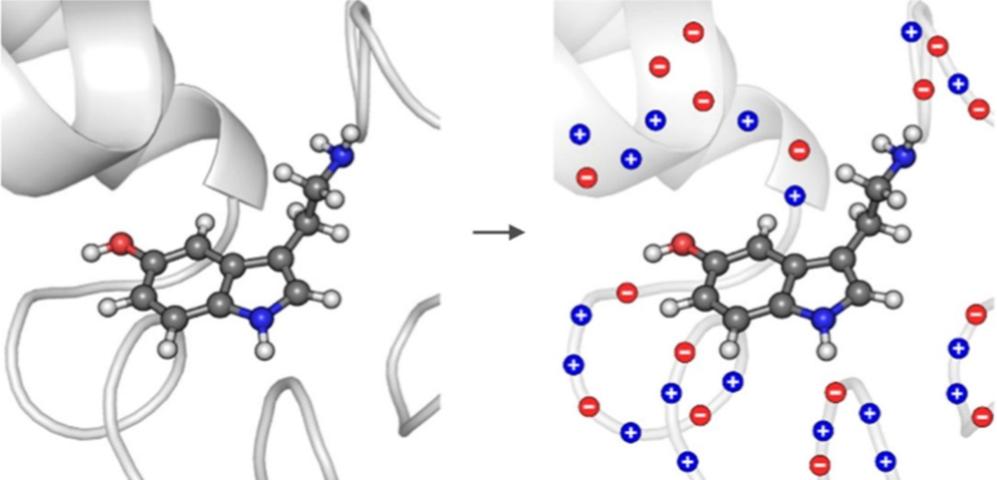
Scheme demonstrating the working principle of our electrostatic embedding QM/MM. The investigated system is divided into two subunits, comprising the reactive moieties (reaction kernel drawn in balls and sticks, QM) and the enzymatic scaffold (MM). The latter is then transformed into a set of discrete point charges, which are included in the quantum-mechanical computations performed for the kernel.

On the downside of our approach, a treatment lacking van der Waals interactions is essentially incomplete, rendering popular protocols such as geometry optimization or MD simulation impossible, thereby restraining its availability to single point calculations accompanied by charge distribution and orbital analysis. Considering this limitation, illustratively, it can be then seen as a kind of emulator that can be used to neatly translate (postprocess) data produced formerly with cost-effective simulation methods (such as MM) into information that would be acquired via high level of theory (QM) computations (therefore, no meaningful output can be generated from a very scratch using solely our methodology). Nevertheless, this treatment has been capable of accurately explaining not only the decisive role of electrostatics in the catalytic function of a selected enzymatic system(s)^16^ but also its regulation of the enzyme’s performance in subtle details.^18,19^

When expanding this approach to other enzymes, our preliminary treatment suggested noteworthy dependence of interaction energies between the RK and its electrostatic environment on the definition of the former, and the computed electrostatic stabilization was sometimes evidently exaggerated (in a minor part of cases, leading even to negative activation energies). This pointed us to an important caveat of our electrostatic embedding approach, which derives from the omission of nonelectrostatic interactions. Namely, neglecting these interactions, Pauli’s repulsion in particular, results in overpolarization of the boundary between the QM and MM parts of the system, especially if the boundary is polar. That is because electron density is polarized by an incorrect, purely attractive potential exerted by positive point charges close to the boundary. This artifact is called electron spill-out as the electron density leaks from the QM region into the MM environment.^14,20^ To avoid it, several approaches were proposed, such as modifying the term for calculating Coulombic interactions,^21^ Gaussian smearing of classical charges,^22,23^ or dampening of electrostatic interactions by pseudopotentials.^20^ In this vein, redistribution of the boundary charges,^24^ as well as incorporation of the tuned link atoms,^25^ both related to “carving” of the QM kernel, should also be mentioned herein. On the other hand, according to literature reports, using more sophisticated embedding schemes explicitly accounting for the nonelectrostatic interactions and their effect on the electron density of the QM region comes at increased computational cost and does not necessarily translate to increased accuracy.^26^

In attempts to efficiently improve our multiscale electrostatic embedding scheme, but at the same time retain its remarkable simplicity, the motivation of the present work is to devise a simple yet effective protocol to mitigate the aforementioned electron spill-out effects. We feel that a distance-based attenuation of the MM charges present near the QM boundary could be used for this purpose. Accordingly, in the present work, we apply and test various scaling schemes for the nearest point charges, as demonstrated below. The advantage of the proposed manipulations is that full compatibility with Gaussian’s input file syntax^17^ is retained at virtually no additional cost.

In the first stage, we devise and test^27^ the improved approach on few molecular pairs consisting of simple ionic entities (Na^+^, OH^–^) and/or water molecule, whereas a more complex treatment includes the following enzymatic systems:

i) MAO-A-catalyzed reaction of serotonin to 5-HIA; Monoamine oxidase A (MAO-A, *EC 1.4.3.4*) is a flavoenzyme that facilitates oxidative degradation of serotonin to 5-hydroxy-3-indoleacetaldehyde (5-HIAL). Unlike most other flavoenzymes, MAO-A contains FAD that is covalently bound to its structure. Misregulations or inactivating mutations of MAO-A are related to various neurological disorders, including, e.g., Brunner syndrome.^28–30^

ii) HisA-catalyzed and PriA-catalyzed reaction of PRA to CdRP; 1-(5-Phosphoribosyl)-5-[(5-phosphoribosylamino)­methylideneamino]­imidazole-4-carboxamide isomerase (HisA, *EC 5.3.1.16*) and *N*-(5′-phosphoribosyl)­anthranilate isomerase (TrpF, *EC 5.3.1.24*) are single-substrate enzymes involved in histidine and tryptophan biosynthesis, respectively. HisA and TrpF catalyze two related isomerization reactions of *N*′-[(5′-phosphoribosyl)-formimino]-5-aminoimidazole-4-carboxamide-ribonucleotide (ProFAR) and *N*-(5′-phosphoribosyl)­anthranilate (PRA) into *N*′-[(5′-phosphoribulosyl)-formimino]-5-aminoimidazole-4-carboxamide-ribonucleotide (PRFAR) and 1-(*O*-carboxyphenylamino)-1-deoxyribulose-5-phosphate (CdRP), respectively. However, in some actinobacteria, the two reactions are instead catalyzed by bisubstrate enzyme phosphoribosyl isomerase A (PriA, *EC 5.3.1.24*), which accepts both ProFAR and PRA as substrates.^31–33^

iii) HBDH-catalyzed reaction of 3-oxovalerate to (R)-3-hydroxyvalerate; Enzyme 3-hydroxybutyrate dehydrogenase (HBDH, *EC 1.1.1.30*) participates in the synthesis of ketone bodies during fatty acid metabolism, where it catalyzes reversible NADH-dependent reaction of (*R*)-3-hydroxybutyrate and acetoacetate.^34^ Alternatively, HBDH catalyzes the conversion of (*R*)-3-hydroxyvalerate and 3-oxovalerate. Due to the stereospecific nature of HBDH catalysis, engineering efforts have been focused to expand the range of accepted substrates, which is relevant, for example, in synthesis of biopolymers.^35–37^

## 2 Computational Details

### 2.1 Software and Resources

All of the computations were performed at the Ažman Computing Center located at the National Institute of Chemistry. The QM/MM calculations were based on the Density Functional Theory (DFT) methodology implemented in the Gaussian 16 (rev. C.02) package.^17^ The choice of the M06-2X functional and the 6-31+G** basis set was dictated by the previous works on the investigated issue.^16,18^ Processing of the input and output files was done in a *Python* environment (v. 3.8) with a particular use of the *NumPy* library (v. 1.21).^38^ The corresponding scripts are provided in the Supporting Information in the form of a ready-to-use routine (G16_CHd.py/sh).

MD simulation snapshots representative of the enzymatic reaction in the reactant and transition state used during the present inquiry originate from the former studies on enzymatic reactions^30,33,37^ and were provided to us either explicitly or in the form of the ready-to-use MD simulation inputs. In the latter case, the aforesaid data files were reproduced according to the enclosed guidelines^33^ with the Q 5.0 package.^39^

### 2.2 Scheme of Computations

#### 2.2.1 Minor Two-Component Systems

At the onset, for the selected minor inorganic entities (H_2_O, OH^–^, and Na^+^), values of the point charges were estimated, for the purpose of which the scheme by Besler–Merz–Kollman was adopted.^40^ In the next step, the aforesaid species were combined pairwise (several different spatial orientations were considered), and for the resultant doublets of molecules, potential energy surface (PES) scans were performed, depicting how the energy of the given doublet evolves as the constituent moieties, treated as quantum objects, move apart from each other. Then, for each point on the PES, the energy of intermolecular interaction was computed by subtracting primary energies computed independently for the individual components, corrected for the basis set superposition error (BSSE), from the energy of the doublet taken from the PES. Subsequently, within all the generated molecular pairs, one of the entities was replaced with its point-charge representation, and the PES scans were rerun following the same scheme of computations (the subtraction operation involved in this case energy of the QM object in and without the presence of the charges, and the energy of self-interacting charges themselves). The latter step was repeated several times, as the values of the point charges were subjected to different scaling schemes, as explained below.

#### 2.2.2 Macromolecular Systems

The evaluation of the proposed electrostatic embedding algorithm, done successively for the four considered enzymatic systems (MAO-A, HisA, PriA, and HBDH, see Figure S3), started with selection of 10 random MD simulation snapshots extracted from 5 distinct, independently equilibrated MD trajectories, depicting the enzyme itself as well as the related substrate in the initial phase of the occurring reaction (reactants, 5 snapshots labeled with letter “R”) and in the phase corresponding to the transition state (5 snapshots denoted as “TS”).^16,18^ Then, based upon identified principal reactive moieties of the corresponding enzymatic reaction (such as substrate–cofactor pairs), the RK subunits were defined for each of the aforesaid samples. Subsequently, these RKs were subjected to slight adjustments, comprising truncation of the selected (redundant) pendants (see Figure S3; to maintain the original singlet multiplicity, all the severed bonds were saturated with hydrogen atoms).^16,18,25^ In brief, such fine-tuning allowed not only minimization of the size of the RKs (computational cost reduction) but also manipulation of their overall charge (feature relevant for the validation process).

Thereafter, for each snapshot, all the residues (including water molecules) within the range of 12 Å from the indicated RKs were one by one carved out from the protein scaffold and paired with the RKs (the spatial coordinates of all of these entities remained unchanged). For each of the doublets generated in this way (both subunits were treated as the quantum objects in vacuum conditions), the interaction energies were evaluated by computing the overall DFT energies of the given molecular pair and then deducting from it the BSSE-corrected constituent energies of the separated components (see the first paragraph of Section 2.2).

It is the final stage, the residues paired with the RKs were eventually replaced with their point-charge representations (as defined in the original MD simulations), and the DFT calculations were rerun several times, each time using different scaling schemes for the point charges (CH). At the very end, the output intermolecular energies were collated against their formerly evaluated counterparts, involving solely a QM description of the analyzed molecular systems.

## 3 Results and Discussion

### 3.1 Minor Two-Component Systems

In order to shed some initial light on the investigated issue outlined in the introductory section, the present study shall begin with a prefatory analysis carried out for a few elementary model systems composed of very core inorganic species, comprising water (H_2_O), hydroxide anion (OH^–^), and sodium cation (Na^+^). In this regard, all the compounds listed above were mutually coupled into pairs (doublets), which were then made to slowly move apart, depicting how interaction energy between the two moieties evolves with the increasing distance. Then, in each of such created pairs, one of the constituent molecules was replaced with a set of point charges, adjusted to mimic the corresponding hydrogen, oxygen, and sodium atoms/ions.^40^ The foregoing energetic profiles were then recomputed and collated with their previously acquired equivalents.

As can be observed on the resultant plots (see Figures 2 and S1), energies of interactions evaluated throughout the purely QM/DFT approach, and according to the point-charge approximation (CH), are reasonably consistent in a wide range of distances. Yet, at the onset, they become essentially incompatible. This is expectable, as at close range, the atoms start to repel each other, which by very definition cannot be reflected by the model comprising only their spaceless point-charge representations. Nevertheless, the low-distance section of these profiles is of less relevance for the purpose of the present technique because even the closest atoms involved in the evaluated nonbonding interactions of our interest are well beyond the highly repulsive region of the intermolecular potential. Accordingly, the main question then arises if the CH model can be efficiently modified to minimize the indicated discrepancy in the ∼2.0–3.0 Å distance range, in which the nearest surrounding atoms typically emerge in the case of reacting moieties embedded in a macromolecular (enzymatic) environment.^41^

**Figure 2:**
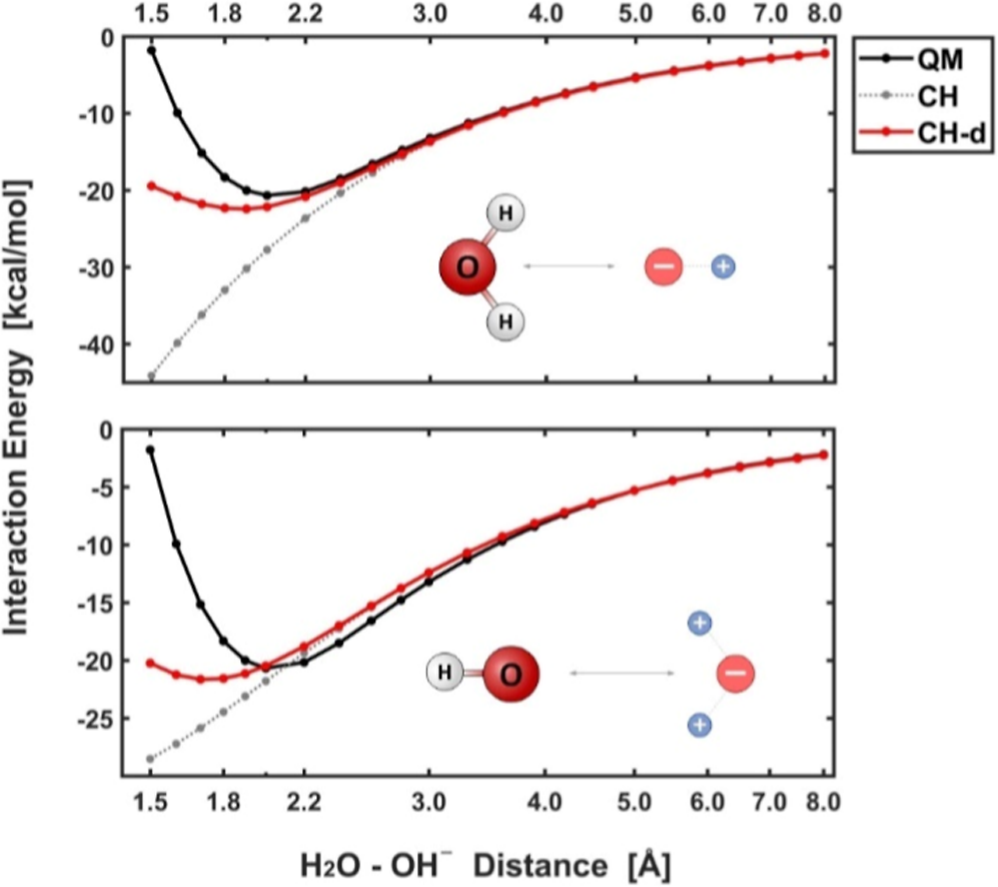
Problem of electrostatic embedding shown on the mutual interaction of H_2_O and OH^–^ molecules (for other doublets, see Figure S1). On the short distances, predictions obtained with the CH model (gray line) remain significantly different from their QM counterparts (black line). Yet, attenuation of the point charges by a distance-dependent damping scheme according to eq 2 (red line, CH-d) in such a region can effectively lead to reduction of the aforesaid discrepancy (cf. Figure S2).

One of the possible solutions for doing so, as commonly used in MD and QM/MM simulations, is to patch the utilized calculus with an additional term (e.g., Lennard-Jones potential, LJ), that would explicitly incorporate the aforementioned repulsion forces into the computations.^11,13,21^ This, however, is a rather nontrivial task that entails considerable research and programming efforts related to parameterization and encoding of the indicated function (in principle, LJ parameters have to be individually defined for each type of interacting atom pairs, which makes a great number of variables; also implementation of the additional repulsion term into the computations requires modification of the very source code of the utilized QM program, which is not always feasible).^21,26^ Therefore, in order to keep our CH model in as a simple and easy-to-use form as possible, rather than explicitly including the repulsive nonbonding terms, we propose a more robust and substantially less demanding protocol to implicitly mitigate the effects of an incomplete model by scaling of the corresponding point charges (CH-d),^41^ focusing primarily on the 2–3 Å distance range on the PES (Figure S2).

To specify, by comparing the PES profiles shown in Figure 2 (and Figure S1), it can be derived that in the aforementioned range of distances, the interaction energies predicted according to the CH approach are typically overestimated compared to the ones stemming from QM computations (Figure 2, bottom graph). Hence, to become more accurate, the CH profiles have to be slightly downscaled, which can be achieved by scaling down the values of the point charges used in the calculations (Figure S2). Naturally, this cannot consist in concurrent damping of all the charges but should be done for each point charge separately—most practically, depending on the distance from the coupled entities with which it does interact.^21,24,41^

For this particular purpose, the proposed form of a distance-dependent scaling scheme is the sigmoidal damping function *d*_(r)_, given by the following formula:

1 $$d_{\left(r\right)}=\frac{1}{1+\exp\left(\frac{r-d_{c}}{d_{w}}\right)}$$

Remarkably, assuming the symmetry of the sigmoidal “ramp”, the two coefficients in the above expression defining, respectively, its center and width, i.e., *d*_c_ and *d*_w_ (for visualization, confer to Figure 3), can be derived from a single quantity, *r*_0_, defining the actual range of charges attenuation (we arbitrarily take the 99% of the asymptotic *d*_(r)_ value as a reasonable approximation of a point *r*_0_ beyond which the charges are effectively unscaled).

$$\begin{array}{l} d_{c}=\frac{1}{2}r_{0}\\ d_{w}=\frac{r_{0}}{2\cdot \ln\left(\frac{1}{1-0.99}\right)}=\frac{d_{c}}{\ln\left(100\right)} \end{array}$$

**Figure 3:**
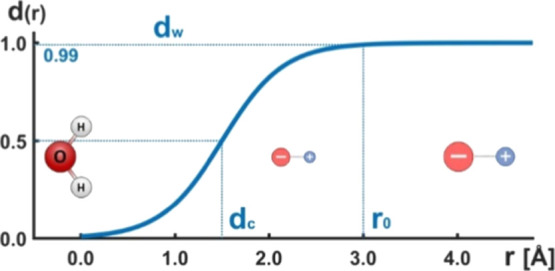
Illustration of the dumping function (eq 2) proposed to attenuate the point charges localized in the immediate vicinity of the RK entity.

Accordingly, the proposed damping function (eq 1) can be eventually constructed with the use of only one parameter, hereinafter referred as to the damping range (or simply the cutoff), that has to be subjected to former optimization (*r*_0_).

2 $$d_{\left(r\right)}=\frac{1}{1+\exp\left(\frac{r-\frac{1}{2}r_{0}}{r_{0}}\cdot \ln\left(100\right)\right)}$$

For the explored two-component samples, the fine-tuning of the indicated *r*_0_ coefficient is rather straightforward and can be done manually for each pair of the considered species. However, for the complex enzymatic systems composed of a number of different interacting moieties, the analogical task becomes more demanding. Yet, taking into account the significant improvement of the accuracy of PES profiles observed for the studied model cases upon application of the proposed charge-damping scheme (see CH-d in Figures 2 and S1), such an effort definitely seems worth to be taken.

### 3.2 Macromolecular Systems

Having addressed the prefatory model cases, in pursuing its very objective, the main focus of the undertaken research shall be eventually shifted toward the macromolecular enzymatic systems. Due to the complexity of such structures, the previous scheme of the analysis has to be adequately modified. Therefore, instead of addressing the myriad of molecular doublets being individually slid apart, a more feasible approach has been adopted. Namely, for all the acquired use-cases, i.e., reactions involving MAO-A, HisA, PriA, and HBDH enzymes,^30,33,37^ the corresponding RKs (see Figure S3) were singled out and systematically coupled one-by-one with all the remaining residues within a radius of 12 Å. Then, for each of the generated pairs of species, energies of interactions were calculated, treating the aforesaid subsidiary residues once as fully quantum objects and once as the discrete array of point charges located at atomic positions (see Figure 1). Consequently, in such a way, a massive set of representative and at the same time well-varied entries have been obtained, believed to allow for carrying out a statistically valid evaluation procedure (the indicated variability covers both diverse conformations taken by the kernels as well as spatial distributions of the surrounding moieties).

Accordingly, in order to cross-reference how the two foregoing computational approaches, i.e., QM and CH, depict interactions between different compounds diversely distributed over space, the corresponding output energies were juxtaposed against each other in the form of the 2D correlation plots (see Figure 4 for the selected case of MAO-A enzyme).^20^ Then, for each pair of the corresponding data sets, Pearson correlation coefficient *r* was determined, together with root-mean-square errors, RMSE (QM energies set as the reference), and the overall sum of the collated intermolecular energies Σ_avg_ *E* (averaged over all the corresponding MD snapshots).^42^ We take *r*, RMSE, and Σ_avg_ *E* as measures for the quality of match between the interaction energies computed by the CH and QM approaches. These quantities are provided in Table 1 for all of the considered enzymes and their corresponding RKs, while correlation plots together with the pertinent *r* and RMSE values are displayed in Figures 4 and S4 for the case of MAO-A.

**Figure 4:**
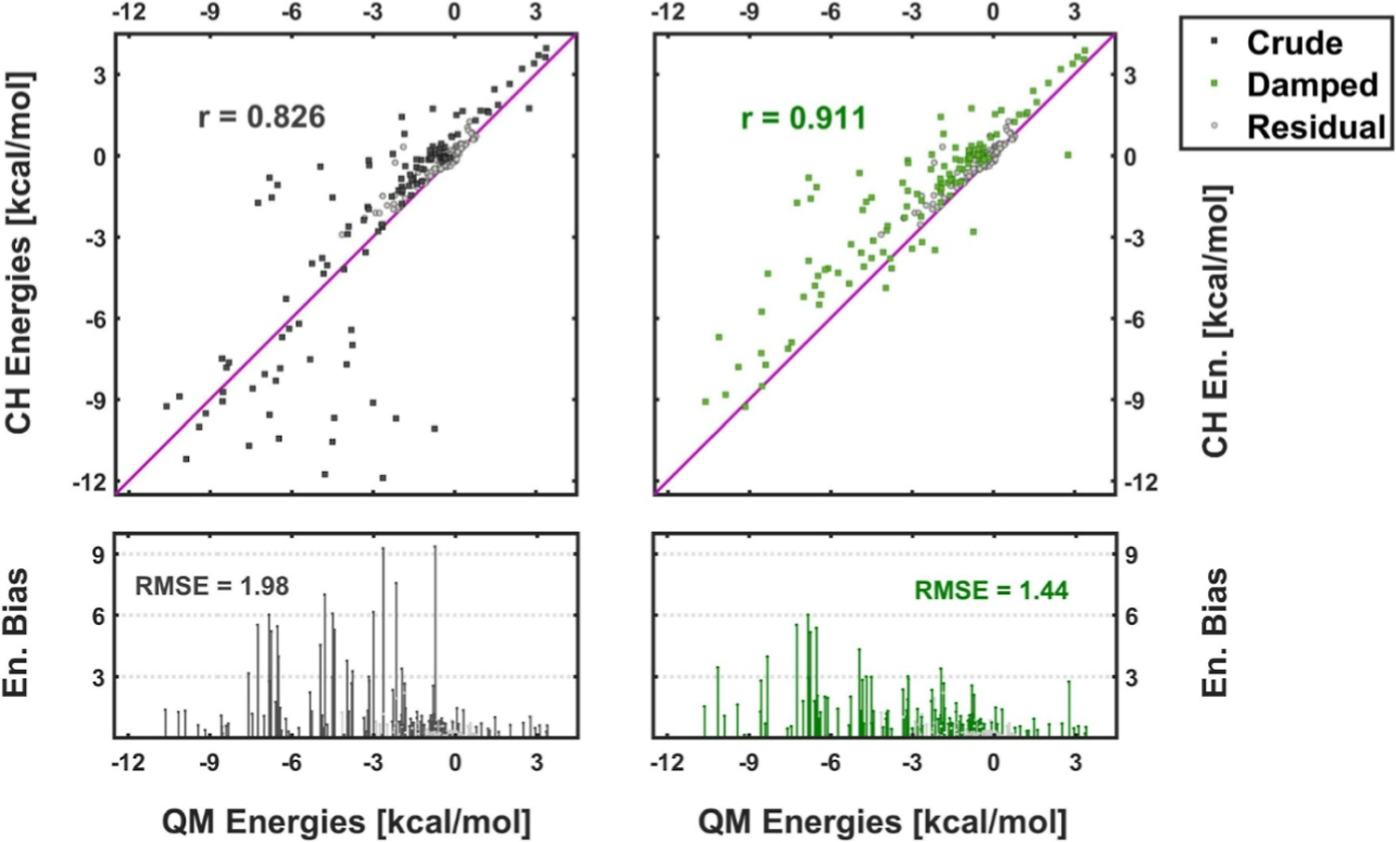
Correlation plots of the interaction energies computed for the MAO-A enzyme (R structures, neutral kernel, *Q*_RK_ = 0) according to the QM and CH methodologies prior to (“Crude”, black dots, panels on the left) and after (“Damped”, green dots, panels on the right) application of the damping function (*r*_0_ = 3.0 Å). Gray dots (“Residual”) represent residues localized beyond the damping range. Panels at the bottom depict offsets between the reference (QM) and CH/CH-d values of the interaction energies. For the summary of statistical quantities (Pearson *r*, RMSE), see Tables 1 and S1. Confer also to Figure S4 for the complementary correlation plots derived for the charged reaction kernel (*Q*_RK_ = −2).

**Table 1:** Summary of the Statistical Quantities Utilized to Assess the Performance of the CH-d Approach for Different Enzymatic Reactions (in Separately Treated R and TS Stages of the Reaction), Differently Charged RKs, and Different Parameter Values of the Charge-Damping Scheme^a^ ^,^ ^b^

quant	R structures					TS structures				
	QM	CH	CH-d			QM	CH	CH-d		
			2.0	2.5	3.0			2.0	2.5	3.0
MAO–A (*Q*_RK_ = 0)										
Σ_avg_ *E*	–78.4	–69.3	–67.2	–59.0	**–45.6**	–83.5	–81.2	–78.1	–67.0	**–49.6**
*r*		0.844	0.859	0.894	**0.914**		0.848	0.864	0.902	**0.915**
RMSE		1.51	1.39	1.13	**1.10**		1.60	1.45	1.11	**1.14**
MAO–A (*Q*_RK_ = −2)										
Σ_avg_E	–272.3	**–211.8**	–210.6	–198.2	–169.0	–270.3	**–221.4**	–220.4	–202.3	–173.4
*r*		**0.992**	0.992	0.990	0.980		**0.993**	0.992	0.988	0.972
RMSE		**3.21**	3.27	3.74	5.16		**2.72**	2.83	3.47	5.03
HisA (*Q*_RK_ = 0)										
Σ_avg_ *E*	–31.6	–36.0	–33.5	**–25.9**	–16.2	–51.9	–61.1	–57.2	**–45.6**	–29.8
*r*		0.910	0.921	**0.946**	0.943		0.866	0.891	**0.929**	0.869
RMSE		1.11	0.93	**0.54**	0.57		1.35	1.12	**0.74**	1.02
HisA (*Q*_RK_ = −2)										
Σ_avg_ *E*	–397.1	**–316.8**	–287.7	–191.0	–58.2	–440.1	**–337.2**	–308.2	–201.4	–47.9
*r*		**0.965**	0.962	0.936	0.846		**0.991**	0.989	0.966	0.880
RMSE		**5.59**	5.92	7.97	11.67		**3.33**	3.78	6.46	10.99
HisA (*Q*_RK_ = +1)^c^										
Σ_avg_ *E*	+25.82	+59.04	+**59.28**	+55.01	+43.28	+20.75	+**41.66**	+31.48	+18.56	+4.63
*r*		0.991	**0.992**	0.985	0.950		**0.985**	0.971	0.933	0.881
RMSE		1.63	**1.44**	1.79	3.21		**1.72**	2.27	3.46	4.69
PriA (*Q*_RK_ = 0)										
Σ_avg_ *E*	–28.6	–37.3	–34.1	–26.6	**–17.4**	–48.3	–60.9	–55.3	**–41.2**	–23.8
*r*		0.702	0.758	0.858	**0.907**		0.929	0.948	**0.973**	0.947
RMSE		1.53	1.30	0.87	**0.67**		1.45	1.14	**0.72**	1.17
PriA (*Q*_RK_ = −2)										
Σ_avg_ *E*	–620.8	**–503.4**	–474.7	–385.3	–246.4	–603.3	**–503.6**	–473.9	–371.1	–223.2
*r*		**0.990**	0.989	0.978	0.939		**0.992**	0.992	0.982	0.945
RMSE		**4.26**	4.71	6.91	11.23		**3.75**	4.16	6.68	11.14
HBDH (*Q*_RK_ = 0)										
Σ_avg_ *E*	–140.1	–130.8	**–122.6**	–100.8	–72.6	–154.7	–143.7	**–128.7**	–101.0	–70.5
*r*		0.990	**0.990**	0.983	0.955		0.987	**0.986**	0.968	0.929
RMSE		1.40	**1.23**	1.54	2.66		1.76	**1.53**	2.15	3.31
HBDH (*Q*_RK_ = −1)										
Σ_avg_ *E*	–287.3	**–258.8**	–243.1	–199.7	–144.0	–310.8	**–279.2**	–252.3	–198.5	–138.6
*r*		**0.996**	0.995	0.983	0.946		**0.994**	0.989	0.960	0.907
RMSE		**1.55**	1.69	3.32	5.80		**1.92**	2.57	4.82	7.30
HBDH (*Q*_RK_ = −3)										
Σ_avg_ *E*	–766.3	**–726.0**	–662.7	–501.9	–317.2	–841.9	**–761.3**	–692.9	–532.0	–343.0
*r*		**0.978**	0.977	0.953	0.895		**0.990**	0.988	0.969	0.917
RMSE		**7.46**	7.65	11.27	16.98		**5.21**	5.73	9.85	15.71

a In addition to the Pearson *r* correlation coefficients and RMSE (eq 2, kcal/mol), also the averaged values of the total summed intermolecular energies are provided (Σ_avg_ *E*, kcal/mol). The best matches among the variants of the CH-d scheme are indicated in bold. For supplementary descriptors, see Table S1.

b Values of the summed up interaction energies (Σ_avg_ *E*, averaged over all snapshots) feature on the average standard deviation of about 10%, reaching a maximum value of 35% for PriA.

c Charge of the reaction kernel *Q*_RK_ = +1 was generated artificially by substituting the negatively charged PO_4_^2–^ fragment with the NH_3_^+^ group (cf. Figure S3).

Looking at the values of the listed numerical indicators (Tables 1 and S1), as well as on the matching graphs (Figure 4), it can be inferred that, in general, agreement between the interaction energies computed throughout the full QM treatment and approximated via unscaled CH embedding is quite reasonable for low interaction energies (within ±5 kcal/mol) but much less so for stronger interactions (Figure 4, graph at the left). Furthermore, the consistency in the computed energies using unscaled charges is better (as displayed on the example of MAO-A but holds similarly also for other enzymes) if the RK is defined as a charged entity (*Q*_RK_ = ±*n*; cf. Figure S4); for neutral cores (*Q*_RK_ = 0; cf. Figure 4), the discussed alignment becomes worse. This feature may be then related to the subsequent observation, that in the neutral RK, the singular interaction energies predicted according to the CH approximation, in a substantial part, tend to be overestimated with respect to their QM equivalents (considered hereinafter as the reference points). This can lead to an incorrect assessment of their importance and, ultimately, to misguided interpretation of the analyzed enzymatic system (see Table S2). Remarkably, the indicated regularity applies mainly to those residues that are in a rather close proximity to the RK (see Figure S5),^16^ which, in principle, complies with the Coulomb law.^21^ In light of the above, it can be expected that the observed discrepancy can be mitigated by adopting the already discussed empirical correction, comprising the attenuation of the point charges used during the CH computations (Section 3.1).

Another feature worth mentioning is the “outlier” set of points displayed in the inset of right-column graphs in Figure 4, featuring very large interaction energies between approximately −120 and −150 kcal/mol. All of these cases correspond to one single charged residue, namely, Arg40, located relatively close (∼9 Å) to the RK, and the interactions are very strong only when the RK is negatively charged; for a neutral RK, the corresponding interactions are well within the range exhibited by other residues, roughly between −10 and +5 kcal/mol. This is an illustrative demonstration of the highly relevant role of electrostatics in enzyme active sites.

Consequently, following the outlined trail, the energies of interactions, serving hitherto as probe quantities,^16^ were recomputed according to the CH-d scaling scheme employing the sigmoidal filter (eq 2), for which several different cutoff parameters (*r*_0_) were specified (cf. Figure 3, variable *r* was defined as the distance between the particular point charge and the nearest atom of the core reactive moieties). At the end, the resultant outcome was subjected to the cross-reference processing, carried out in an analogical manner as in the primary initial case. For MAO-A, analogous correlation plots as obtained with unscaled charges are displayed in the right panels of Figures 4 and S4. In addition, Figure 5 displays variations in the *r* coefficient as well as in the RMSE of the CH-d energies as a function of the value of the damping parameter *r*_0_ (cf. eq 2 and Figure 3).

**Figure 5:**
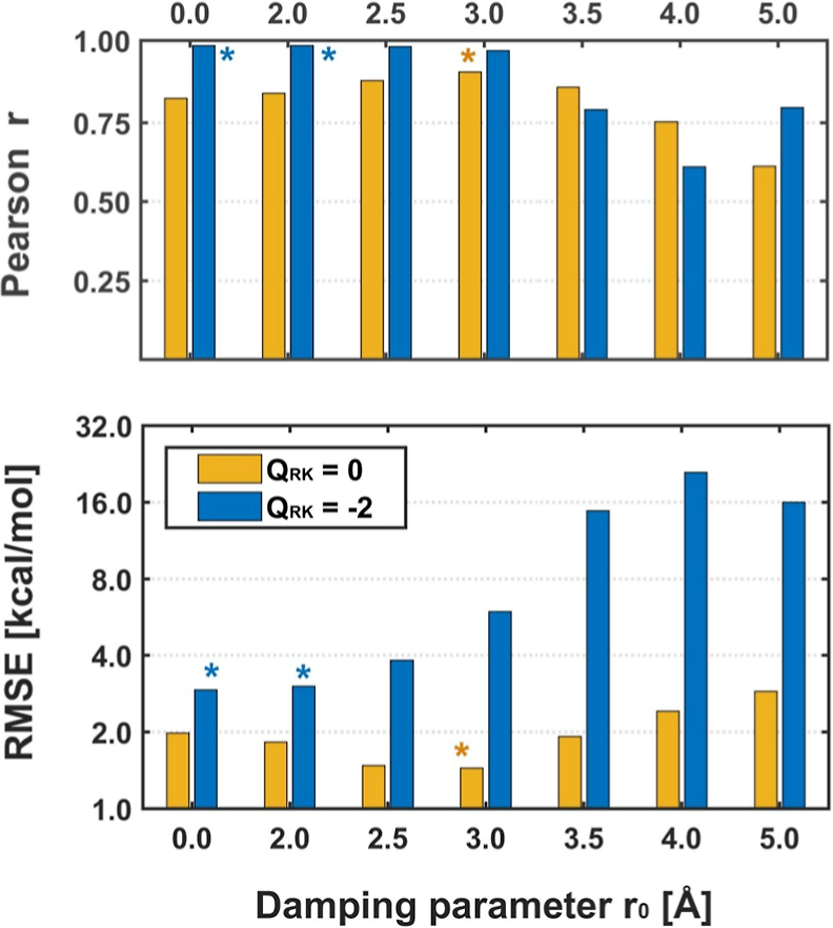
Accuracy (and precision) of the interaction energies predicted for the MAO-A enzyme with the CH-d model, dependent on the adopted damping parameter (*r*_0_ in eq 2). For each value of *r*_0_, the three bars correspond, respectively, to the neutral RK (*Q*_RK_ = 0, yellow, Figure 4) and its charged variant (*Q*_RK_ = −2, blue, Figure S4). The colored asterisks (*) indicate the optimal values of the Pearson *r* and RMSE indicators (equal to their maximum and minimum, respectively).

The conclusions stemming from the performed analysis appear to be pretty illustrative (see Tables 1 and S1 and S2 as well as Figures 4, S4, and 5). Namely, attenuation of the point charges indeed allows enhancement of both accuracy and precision of the results obtained with the CH approximation (increase of *r* to over 0.9, reduction of RMSE even up to 50%), but its efficiency strongly depends on the adopted cutoff parameter (Figure 5). Specifically, for the neutral RKs, the optimal value of the damping range, indicated by the highest Pearson’s *r* and the lowest RMSE, varies in the range of 2.5–3.0 Å. Beyond that point, the correction being evaluated is found to become less effective, eventually leading even to worsening of the original results (*r*_0_ > 4.0 Å cf. Table S1; for visualization, see also Figure 4).^41^ Nevertheless, by optimization of damping parameters, a substantial increase in the reliability of the predicted interactions can be achieved, allowing for proper understanding of the very nature of the explored enzymatic system(s) (see Table S2 and the individual interactions listed therein). Just to briefly outline, for the examined enzymatic systems, the most significant alternations stemming from attenuation of the charges are observed for the water molecules surrounding the very reaction kernels (due to their high polarity and proximity to the RKs, the indicated entities incorporated into the very structure of the protein are observed to manifest particularly high interaction energies). On the other hand, the shifts in interaction energies of the protein fragments, except for a few cases (cf. Table S2), are much smaller, becoming practically negligible for more distant residues (which paradoxically can be seen as favorable since some interactions of the latter moieties tend to be underestimated from the outset).

As regards the RKs consisting of charged moieties, the gainful damping range is significantly shorter than in the case of their neutral equivalents and does not exceed 2.0 Å. Considering the individual intermolecular interactions, it can also be pointed out that they are characterized by a significantly wider range of energies. To specify, the average energetic windows comprising the majority of the processed entries remain approximately two times bigger than in the case of neutral RKs (compare Figure 4 with Figure S4). Yet, there are also several data points that protrude far beyond the indicated frame (these are identified to stem from attraction of the positively charged Arg40 side chain with the negatively charged RK; see the inset in Figure S4). Since the latter can overwhelm other interactions (see Table S1), they were decided to be optionally expelled from the performed statistic evaluation (letters F and T in Table S1 denote, respectively, the analysis performed for the full and truncated data sets).

Against the background presented above, it can be concluded that the CH-d damping scheme is not fully universal and has to be optimized and used with proper consciousness. Yet, in return, it can make the CH computations notably more precise and thus more reliable. Along the same lines, it can be also inferred that the results stemming from the foregoing type of calculations should allow for a faithful qualitative description of the explored systems (Pearson *r* greater than 0.90). Still, at the very end of the validation procedure, the quantitative merit of such predictions can be more thoroughly assessed. Thereby, we once again collated the foregoing energies of interactions estimated throughout the CH­(-d) approaches against their QM counterparts, this time with particular emphasis put on the match in energy characterized by Σ_avg_ *E* and RMSE quantities.

Starting with the RMSE values, the estimated average error of the component energies calculated for the individual residues varies by around 1 and 5 kcal/mol, respectively, for the systems with neutral and charged RKs (for the latter, absolute energies of interactions remain significantly higher, see Table 1). This on the other hand translates into approximately 1–2% of the overall interaction energies (Σ_avg_ *E*) determined for such systems, which in general remains a relatively small value (which still can be significant in some cases, such as an in-depth comparative analysis of single intermolecular interactions, see Table S2).^43^

On the other hand, as regards to the above indicated Σ_avg_ *E* quantities, practically in each case (except from the rather unusual positively charged (*Q*_RK_ = +1) HisA system, see the footnote to Table 1), the values estimated throughout the CH-d approach remain underestimated with respect to QM calculations. In terms of absolute values, such discrepancies, akin RMSE, are higher for the charged RKs and, interestingly, for the structures corresponding to the TS, for which the overall interaction energies are observed to be of higher magnitude (while the former regularity stems from the Coulomb law, the latter tends to remain in compliance with the preorganized electrostatics theory mentioned in the introductory part^8,9,16^ ). Yet, being expressed as relative errors, these are paradoxically slightly higher for the neutral core systems, which can be related to the magnified attenuation of the charges applied therein (cf., Figure 5 and Table 1).

Nevertheless, by treating the evaluated energies as a training set, it is possible to derive an effective ad hoc correction, which can allow enhancement of the accuracy of the evaluated CH-d method through the following linear scaling operation:^43^

3 $$E_{QM}\approx \lambda \cdot E_{CH}$$

Although the optimal scaling factor λ may be simply estimated by collating the overall interaction energies stemming from QM and CH-d computations, yet we decided to address the foregoing task in a slightly more sophisticated but in our opinion also more reliable manner (avoidance of a questionable additivity assumption and propagation of the summation error).^16^ Namely, the coveted values of the λ parameter, per analogy to Pearson *r* coefficients, were determined throughout the linear regression (eq 3) performed individually for each of the juxtaposed validation data subsets and then eventually subjected to the appropriate averaging.

Consequently, by analysis of the resultant findings (Table 2), it can be concluded that, quite unexpectedly, for the CH-d computations incorporating zero damping, as well as those comprising attenuation of charges in the narrow range of 2.0 Å, the scaling of the outcome interaction energies is not statistically justified (λ = 1.0). On the other hand, if the adopted cutoff threshold equals *r*_0_ = 2.5 and *r*_0_ = 3.0, the indicated energies should be scaled by the factor of approximately λ = 1.1 and λ = 1.3, respectively. While the indicated amplitudes of λ factors might appear to be slightly underwhelming (their application may not fully reproduce the QM values of Σ_avg_ *E*), they eventually shall allow increasing the accuracy of the foregoing quantities without the risk of infringing their mutual consistency.^27^ Just to indicate, the latter feature tends to be crucial specifically for those types of analyses in which the foregoing interaction energies (or the secondary quantities derived on their basis) are to be mutually compared with respect to the absolute values. Hence, the discussed correction seems not to be of the highest relevance with regard to the solely qualitative investigations.

**Table 2:** Values of the Scaling Coefficients λ^a^ (eq 3) Determined for All the Processed Data Sets by the Linear Regression Procedure

enzyme	*Q*_RK_	R structures				TS structures			
		CH	CH-d			CH	CH-d		
		0.0	2.0	2.5	3.0	0.0	2.0	2.5	3.0
MAO-A	0	1.02	1.04	1.15	1.40	0.97	0.99	1.13	1.45
	–2	1.11	1.11	1.14	1.22	1.09	1.09	1.11	1.17
HisA	0	0.72	0.78	0.96	1.37	0.86	0.90	1.09	1.55
	–2	1.13	1.17	1.32	1.57	1.09	1.12	1.23	1.43
	+1	0.94	0.97	1.04	1.15	0.98	1.02	1.05	1.09
PriA	0	1.03	1.04	1.10	1.26	0.89	0.93	1.08	1.39
	–2	1.05	1.07	1.16	1.32	1.04	1.07	1.17	1.36
HBDH	0	0.93	0.96	1.07	1.28	0.90	0.96	1.11	1.35
	–1	0.99	1.02	1.11	1.26	0.98	1.02	1.14	1.31
	–3	1.01	1.04	1.14	1.28	1.01	1.04	1.14	1.29
mean		**0.99**	**1.02**	**1.12**	**1.31**	**0.98**	**1.02**	**1.13**	**1.34**
STD		0.11	0.11	0.09	0.12	0.08	0.07	0.05	0.13

a Values of standard errors (STD) determined individually for each λ remain on average at the level of 1.5%, reaching a maximum value of 4%.

With the above respect, it can be additionally pointed out that Table 2 reveals noteworthy variations in the scaling factor λ among the considered use-cases of enzymatic reactions, that is, variations exist not only between different enzymes but also between different representations of the reacting moiety of the same enzymatic reaction. This implies that despite the present ability to significantly enhance the accuracy of the estimated electrostatic interactions between the reacting moiety and its enzymatic surroundings, each given case may require its own optimization of the fine-tuning of the electrostatic environment in which the reaction is embedded. With this respect, at the very end of the above discussion, we present the suggested template for calibrating the proposed CH/CH-d protocol, allowing for its case-specific application (which is highly encouraged by the authors).

The indicated scheme starts with selecting a bunch of well-varied snapshots (optimally coming from independent simulation replicas), depicting the system to be investigated at its different (micro)­states. Then, the reaction kernel and principal surrounding moieties have to be defined (which can be streamlined by adopting, e.g., the proposed distance-based criterion) and one-by-one collated against each other. In the next step, for such created bimolecular pairs, reference (QM) and approximated (CH-d) interaction energies need to be computed using different values of damping parameter *r*_0_ (the exemplary ready-to-use inputs provided in Supporting Information may be particularly useful for this purpose). Eventually, the resultant entries have to be subjected to a linear regression procedure (QM vs CH-d), that will concurrently produce values of statistical indicators (*r* and RMSE) and corresponding scaling factor λ, pursuant to the former of which the optimal value of the damping range (*r*_0_) should be selected (indicated by, respectively, maximum of *r* and minimum of RMSE). Incidentally, upon application of some basic-level scripting, practically all the aforementioned steps can be quite easily automated, making the entire procedure relatively effortless for the user of the standard QM software (still, manual verification of the intermediate outputs is highly recommended).

## 4 Conclusions

The principal goal of the present work was to take a closer look at the cornerstones and performance of the discussed electrostatic embedding multiscale algorithm,^16^ developed with a particular view to explore the factors standing behind the catalytic activity of enzymes. In a nutshell, its key concept claims that the sought information may be acquired in a cost-efficient way by running high-level QM calculations that operate on the data formerly produced by more robust MM simulations. The principle of operation is then founded on the ingenious premise that the enzymatic system can be effectively depicted as an envelope made up of a discrete set of point charges (MM), which interacts with an explicitly defined reaction core (QM). While such approximation allows us to substantially reduce the cost and complexity of computations as compared to the full-scale QM/MM simulations, in principle, its accuracy may be called into question, particularly due to artifacts cast onto the electronic structure of the QM subsystem, invoked by the simple point-charge representation of its closest surroundings. Thereby, in the present study, the merit of the foregoing model has been submitted to the in-depth critical evaluation.

At the onset, the reviewed approach was validated by using a few simple and fundamental model systems for evaluation of the reference intermolecular potential energy functions (computed entirely by QM) and confronted with the CH protocol. By collating the resultant quantities, it was concluded that both QM and CH methods provide consistent interaction energies at sufficiently large separations (*r* > 3 Å), but they start to substantially diverge at shorter distances. In order to reduce the observed divergence, an empirical correction to the original CH algorithm was proposed in which the point charges were subject to a one-parameter sigmoidal attenuation, depending on their distance from the closest QM atom. The modified algorithm is designated as CH-d.

In the second step, the performance of the CH/CH-d algorithm was tested on four actual enzymatic systems. Like in the previous case, the evaluation procedure consisted of determination of intermolecular energies coupling the RK with a number of surrounding residues, which was carried out according to both QM and CH/CH-d treatment. Based on the acquired results, it has been concluded that while the “bare” CH algorithm already quite reasonably reproduces the interaction energies obtained by reference QM calculations, its accuracy can be considerably improved by the charge-damping approach implemented in the CH-d algorithm (eq 2). However, damping should be used in a conscious manner, depending on the nature of the reacting moiety (RK), with the net charge of the reaction kernel being one of the most relevant factors. To specify, for the neutral kernels (*Q*_RK_ = 0), attenuation of the charges was observed to be most effective for the damping parameters *r*_0_ set to 2.5 and 3.0 Å. On the contrary, for the charged kernels (*Q*_RK_ = ±*n*), the latter parameter should be reduced so as not to exceed 2.0 Å. In brief, the foregoing guiding principles can be summarized via the following scheme:

$$\begin{array}{l} Q_{RK}=0\to r_{0}=2.5-3.0\to \lambda =1.1-1.3\\ Q_{RK}=\pm n\to r_{0}\leq 2.0\to \lambda =1.0 \end{array}$$

Considering its quantitative accuracy, it must be admitted that the CH-d algorithm being evaluated tends to deviate from perfection. Namely, the absolute values of the interaction energies are generally found to be slightly underestimated, with respect to their QM equivalents. Yet, such divergency can be a posteriori reduced by rescaling of the output quantities with the empirically derived factor λ (eq 3). At the very end, it is worth emphasizing that the values of both *r*_0_ and λ parameters indicated in the above scheme are of rather generalized heuristic character. Thus, one is highly encouraged to individually tune them to best-match the analyzed macromolecular system, the procedure of which can be achieved with a relatively little effort according to the provided guidelines. In addition, further tweaks of the approach such as alternative carving schemes of the QM kernel^25^ or redistribution of the boundary charges^24^ can possibly provide further enhancements, the matter of which represents challenges for the future work.

Consequently, in light of all of the invoked arguments, the validated QM/MM approach, comprising electrostatic embedding of the RK, offers an effective and easy-to-use alternative for studying multiscale electrostatic interactions and their effects on chemical reactions involving enzymes. Into the bargain, the presented charge-damping scheme, elegant in its simplicity, may potentially contribute to development of the quantum-chemical apparatus designed for exploration of such and other complex macromolecular systems. For instance, in the QM/MM algorithm implemented in the Gaussian package (ONIOM),^17,44^ to avoid the electron spill-out effect, there is a built-in option to scale down the point charges in the nearest vicinity of the QM region, which, however, involves predefinition of bonds. In this respect, the proposed approach to adjust the charges via the simple-to-use distance criterion may offer a worth-to-consider alternative.

## Supplementary Material





## Data Availability

Part of data that supports the findings of this study are available within the article and the Supporting Information. A script facilitating implementation of electrostatic embedding for the use with Gaussian software is available in the Supporting Information. Full data is available from the authors upon a reasonable request.

## Notes

Energies of interactions between H_2_O–Na^+^ and H_2_O–H_2_O doublets; impact of the point-charge values onto the interaction energies; depiction of the considered enzymatic systems; correlation plots derived for the charged variant of the MAO-A enzymatic system kernel; dependence of the interaction energies on the distances; secondary descriptors characterizing performance of the CH-d approach; and selected interactions between the reaction kernel and surrounding moieties (PDF)
Script for incorporating the proposed charge-damping scheme into the computations carried out with Gaussian software (G16_CHd.py/sh) (ZIP)

## Notes

The authors declare that no generative artificial intelligence (AI) technologies have been employed in the writing process of this article.
