# Supplementary material for: Rescaling of Point Charges as a Way to Improve the Simple-to-Use Electrostatic Embedding Scheme Developed to Explore Enzyme Activity with QM-Oriented Software
Source: J Chem Inf Model. 2025 Aug 7;65(16):8653–63. doi: 10.1021/acs.jcim.5c01235 (PMC12381855; doi:10.1021/acs.jcim.5c01235)
Supplement: Supplementary file 1 [file ci5c01235_si_001.pdf]

SUPPORTING INFORMATION

FOR

Rescaling of point charges as a way to improve the simple-to-use electrostatic embedding scheme developed to explore enzyme activity with QM-oriented software

Andrzej J. Kałka,[a,b] Aleš Novotný,[a] Jernej Stare [a]*

a) National Institute of Chemistry, Theory Department
Hajdrihova 19, 1000 Ljubljana, Slovenia

b) Jagiellonian University, Faculty of Chemistry
Gronostajowa 2, 30-387 Cracow, Poland

*Corresponding author: *jernej.stare@ki.si*

Co-authors: *andrzej.kalka@uj.edu.pl*; *ales.novotny@ki.si*

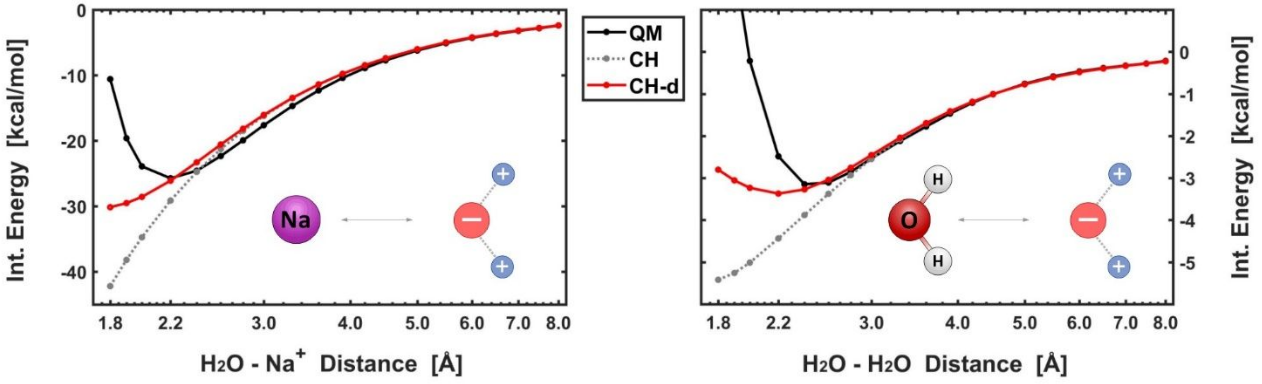

Figure S1. Interactions energies between H_2O molecule and Na^+ cation (left) as well as between two H_2O molecules (right), computed according to QM, CH and CH-d approaches (for more detailed description confer to ***Figure 2***).

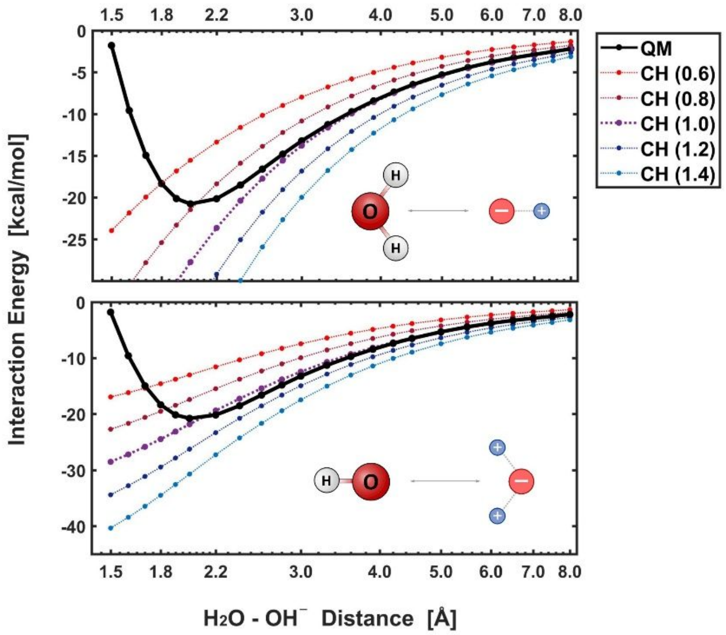

Figure S2. Energies of interactions between H_2O and OH^- molecules computed according to QM and CH methodologies. In the latter case, the resultant energy remains strongly dependent on the values of point-charges specified during calculations (values in parentheses), which can be utilized to increase the accuracy of the output (see ***Figure 2***).

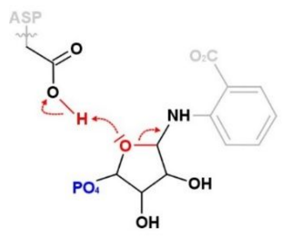

HisA / PriA (ASPH) --- PRA

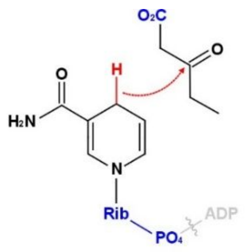

HBDH (NADH) --- 3-oxovalerate

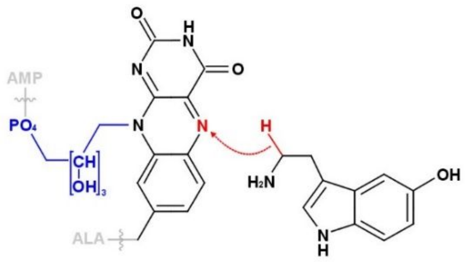

MAO-A (FAD) --- serotonin

Figure S3. Illustration of the enzymatic systems utilized for validation of the proposed QM/MM algorithm comprising damping of point-charges. The reactions occurring therein are presented schematically with red arrows (left: proton transfer from ASPH cofactor to PRA substrate; right: hydride transfer from NADH cofactor to 3-oxovalerate substrate; bottom: hydride transfer from serotonin substrate to FAD cofactor). With blue color are indicated the pendants, neglection of which allows to manipulate the overall charge of the sample. In gray, the redundant fragments neglected during the computations are marked.

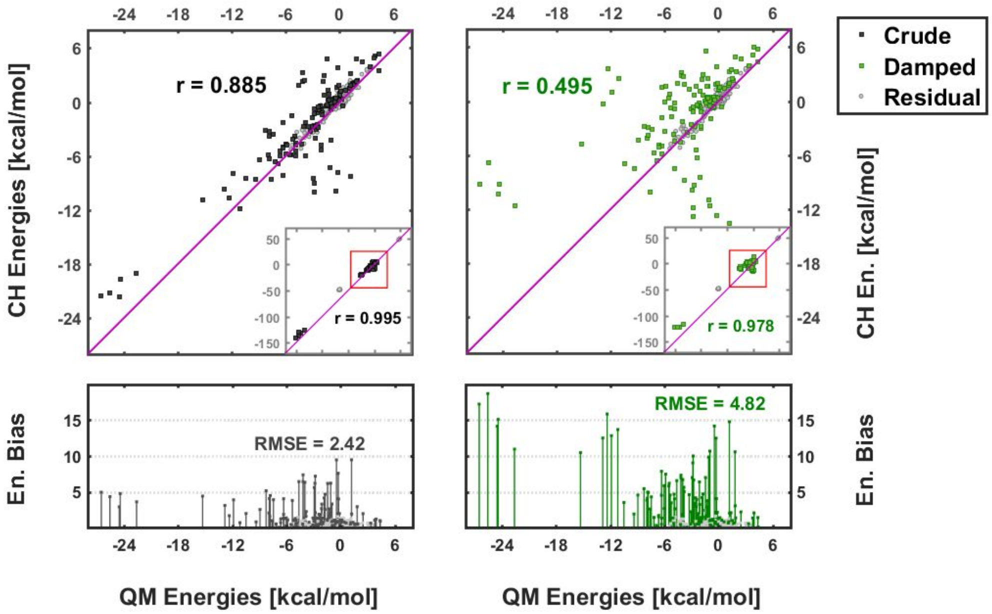

Figure S4. Correlation plots of the interaction energies computed for the charged variant (Q_{RK} = –2) of the MAO-A enzymatic system kernel (cf. ***Figure 4***), according to the QM and CH methodologies prior to ('Crude') and after ('Damped') application of the damping function (r_{cut} = 3.0 Å). Gray dots ('Residual') represent residues localized beyond the damping range. The insets depict the points localized outside the main panels (red windows), representing in particular arginine (Arg40) moieties. Panels at the bottom depict offsets between the reference (QM) and computed CH/CH-d interaction energies. For the summary of statistical quantities (Pearson *r*, *RMSE*) see ***Table 1*** and ***Table S1***.

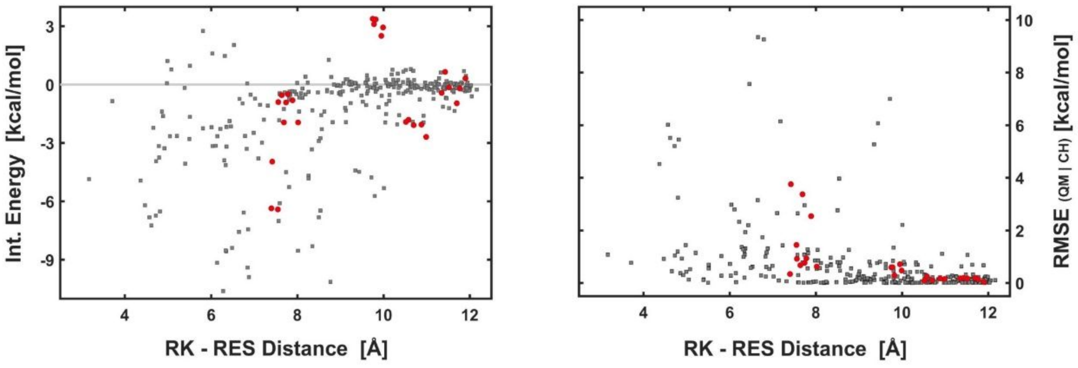

Figure S5. Dependance of the computed (QM) interaction energies on the distances separating individual residues from the reaction kernel (red dots indicate the charged residues) shown on the example of MAO-A enzyme (R structures, c.f. ***Figure 4***). In the right panel, discrepancies (RMSE) in the latter energies computed according to QM and CH approaches are depicted.

Table S1. Supplementary statistical quantities utilized to assess the performance of the evaluated CH-d approach. In contrast to their equivalents provided in ***Table 1***, the indicators presented below are derived only for those residues, whose point-charges were actually attenuated by the adopted damping function. For deciphering of the used abbreviations confer to ***Table 1*** and ***Figure 3***. All energy values are given in kcal/mol.

	**R-structures**							**TS-structures**						
	**QM**	**CH**	**CH-d**					**QM**	**CH**	**CH-d**				
			**2.0**	**2.5**	**3.0**	**3.5**	**4.0**			**2.0**	**2.5**	**3.0**	**3.5**	**4.0**
**MAO-A: Q_{RK} = 0**														
Σ_{avg} E	-75.0	-66.9	-64.8	-56.6	-43.2	-29.1	-18.0	-80.2	-78.7	-75.7	-64.5	-47.1	-30.2	-18.4
E_{min}	-10.6	-11.9	-11.1	-10.4	-9.3	-8.8	-7.9	-12.3	-13.3	-11.9	-10.3	-10.2	-9.9	-9.1
E_{max}	3.4	4.0	4.0	3.9	3.9	3.9	4.0	3.1	3.5	3.5	3.5	3.5	3.7	4.7
r	-	0.826	0.843	0.884	0.911	0.862	0.753	-	0.828	0.846	0.891	0.910	0.814	0.670
RMSE	-	1.98	1.83	1.48	1.44	1.92	2.42	-	2.06	1.87	1.42	1.47	2.12	2.65
**MAO-A: Q_{RK} = -2 (T)**														
Σ_{avg} E	-103.2	-74.8	-73.7	-64.2	-43.8	12.2	55.9	-118.1	-92.8	-91.8	-75.1	-51.4	19.0	5.6
E_{min}	-26.5	-21.6	-20.9	-17.3	-14.6	-16.3	-18.4	-23.8	-22.3	-20.4	-15.6	-15.7	-18.5	-18.5
E_{max}	4.4	0.0	0.0	-9.3	-10.0	-10.5	-12.2	3.1	8.4	8.4	9.0	10.9	116.9	18.4
r	-	0.885	0.873	0.789	0.495	0.013	-0.075	-	0.875	0.851	0.715	0.317	-0.191	-0.326
RMSE	-	2.42	2.53	3.19	4.82	13.76	19.47	-	2.46	2.65	3.60	5.56	13.13	9.42
**MAO-A: Q_{RK} = -2 (F)**														
Σ_{avg} E	-246.3	-207.6	-206.5	-194.0	-164.8	-95.3	-35.4	-250.4	-216.8	-215.8	-197.7	-168.8	-87.6	-91.0
E_{min}	-151.2	-141.2	-141.2	-135.5	-123.1	-111.2	-98.9	-140.7	-132.8	-132.6	-129.7	-121.7	-111.8	-101.9
E_{max}	4.4	0.0	0.0	-9.3	-10.0	-10.5	-12.2	3.1	8.4	8.4	9.0	10.9	116.9	18.4
r	-	0.995	0.994	0.991	0.978	0.792	0.609	-	0.993	0.992	0.986	0.965	0.796	0.874
RMSE	-	2.93	3.02	3.82	5.96	14.77	20.98	-	2.77	2.95	3.89	6.03	13.69	11.10
**HisA: Q_{RK} = 0**														
Σ_{avg} E	-30.8	-35.8	-33.2	-25.7	-15.9	-8.1	-3.8	-49.7	-60.1	-56.2	-44.6	-28.8	-15.7	-7.9
E_{min}	-13.7	-26.2	-23.5	-17.4	-11.0	-7.1	-5.1	-11.3	-16.8	-13.5	-10.6	-7.6	-7.6	-7.6
E_{max}	2.9	2.9	2.9	2.8	2.8	3.7	4.9	3.8	3.7	3.6	2.9	2.7	3.8	4.6
r	-	0.908	0.919	0.944	0.943	0.827	0.660	-	0.851	0.879	0.919	0.856	0.567	0.305
RMSE	-	1.58	1.32	0.77	0.81	1.32	1.65	-	1.95	1.62	1.06	1.47	2.31	2.82
**HisA: Q_{RK} = -2 (T)**														
Σ_{avg} E	-357.4	-291.3	-265.2	-176.1	-51.8	61.9	126.7	-380.6	-322.8	-296.1	-197.1	-55.8	77.6	152.8
E_{min}	-45.2	-39.2	-34.9	-23.8	-30.8	-37.7	-37.6	-45.2	-38.1	-35.3	-26.8	-32.9	-33.4	-29.0
E_{max}	4.8	6.0	6.0	9.1	30.8	54.9	64.4	4.7	5.8	5.8	5.8	32.4	58.3	69.7
r	-	0.979	0.976	0.802	0.119	-0.253	-0.359	-	0.964	0.953	0.751	0.042	-0.335	-0.449
RMSE	-	2.61	3.40	7.60	13.67	18.72	20.86	-	3.01	3.76	7.96	14.42	20.04	22.58
**HisA: Q_{RK} = -2 (F)**														
Σ_{avg} E	-391.4	-321.7	-292.6	-195.9	-63.1	56.2	121.8	-439.3	-346.4	-317.4	-210.6	-57.1	86.7	167.8
E_{min}	-127.2	-119.5	-112.4	-97.0	-85.2	-76.3	-77.2	-143.4	-129.9	-127.7	-119.7	-107.9	-98.6	-94.2
E_{max}	80.9	79.3	79.3	79.3	79.3	79.3	79.4	76.7	81.3	81.3	81.3	81.3	81.3	81.4
r	-	0.960	0.958	0.931	0.839	0.720	0.671	-	0.991	0.990	0.967	0.882	0.769	0.712
RMSE	-	7.72	8.18	11.05	16.21	20.79	22.68	-	4.46	5.07	8.74	14.91	20.36	22.86
**HisA: Q_{RK} = +1 (T)**														
Σ_{avg} E	64.3	92.7	89.8	77.8	58.1	34.4	14.8	44.5	58.1	58.3	53.3	42.2	24.8	11.1
E_{min}	-7.5	-9.5	-6.9	-7.0	-13.5	-17.5	-17.0	-8.8	-18.9	-13.6	-13.6	-24.2	-27.9	-27.0
E_{max}	10.3	12.8	10.7	9.5	12.6	20.5	22.5	13.3	14.7	11.5	9.1	17.7	23.4	22.4
r	-	0.851	0.828	0.666	0.264	-0.014	-0.137	-	0.831	0.828	0.602	0.175	-0.010	-0.078
RMSE	-	1.92	1.92	2.43	3.90	5.31	5.93	-	2.45	2.17	3.06	5.31	6.74	6.99
**HisA: Q_{RK} = +1 (F)**														
Σ_{avg} E	35.3	59.6	59.9	55.6	43.9	24.8	6.1	33.9	45.6	35.4	22.5	8.6	-8.2	-21.4
E_{min}	-77.3	-91.0	-84.3	-68.9	-54.1	-51.8	-51.9	-54.2	-56.7	-56.7	-56.7	-56.7	-56.7	-56.8
E_{max}	44.0	45.1	45.1	44.8	43.7	41.8	40.4	45.2	46.4	46.4	46.4	46.4	46.2	45.5
r	-	0.990	0.991	0.983	0.942	0.881	0.845	-	0.982	0.966	0.920	0.859	0.825	0.825
RMSE	-	2.27	2.00	2.50	4.54	6.32	7.16	-	2.42	3.22	4.93	6.69	7.58	7.55

Table S1. - *continuation*

	R-structures							TS-structures						
	QM	CH	CH-d					QM	CH	CH-d				
			2.0	2.5	3.0	3.5	4.0			2.0	2.5	3.0	3.5	4.0
**PriA: Q_{RK} = 0**														
Σ_{avg} E	-29.2	-38.1	-35.0	-27.5	-18.2	-11.1	-6.6	-50.1	-62.9	-57.3	-43.2	-25.9	-12.5	-5.0
E_{min}	-13.6	-14.4	-14.4	-14.4	-14.1	-13.5	-12.3	-27.7	-30.2	-28.5	-25.1	-23.2	-20.4	-17.1
E_{max}	4.1	4.3	4.3	4.1	3.8	4.1	4.5	13.9	13.5	13.1	11.8	9.8	7.8	6.6
r	-	0.685	0.743	0.850	0.905	0.854	0.769	-	0.925	0.946	0.972	0.947	0.847	0.748
RMSE	-	2.35	1.99	1.33	1.03	1.29	1.58	-	2.23	1.75	1.10	1.80	2.77	3.34
**PriA: Q_{RK} = -2 (T)**														
Σ_{avg} E	-280.7	-238.5	-218.0	-146.9	-45.1	47.3	101.0	-273.7	-231.7	-211.7	-141.8	-44.5	43.2	91.7
E_{min}	-33.2	-30.0	-30.0	-33.7	-43.1	-43.6	-37.5	-34.2	-29.2	-27.6	-35.8	-42.2	-42.3	-37.7
E_{max}	11.4	13.0	13.0	13.0	23.3	31.5	40.2	11.8	13.5	13.5	13.5	23.8	32.3	33.2
r	-	0.940	0.898	0.556	-0.009	-0.273	-0.365	-	0.954	0.922	0.649	0.086	-0.227	-0.350
RMSE	-	3.27	4.29	8.50	14.27	18.51	19.98	-	2.91	3.83	7.68	13.13	17.29	18.67
**PriA: Q_{RK} = -2 (F)**														
Σ_{avg} E	-626.3	-528.7	-500.0	-410.6	-271.7	-137.9	-51.5	-632.5	-546.9	-516.7	-412.8	-264.7	-125.8	-37.7
E_{min}	-230.1	-236.6	-230.7	-210.6	-181.1	-144.7	-121.1	-238.3	-241.5	-233.1	-206.6	-178.0	-148.5	-124.6
E_{max}	83.6	88.7	88.7	88.7	88.7	88.7	86.9	74.6	79.6	79.6	79.6	79.6	79.6	79.1
r	-	0.990	0.989	0.978	0.940	0.882	0.840	-	0.992	0.992	0.982	0.945	0.885	0.838
RMSE	-	6.17	6.84	10.10	16.48	22.39	25.91	-	5.30	5.86	9.59	16.16	22.25	26.01
**HBDH: Q_{RK} = 0**														
Σ_{avg} E	-71.4	-58.8	-52.6	-36.4	-16.1	-0.2	7.6	-84.1	-67.5	-55.2	-34.0	-12.2	3.8	12.1
E_{min}	-16.5	-15.8	-14.8	-13.2	-9.2	-9.0	-8.7	-26.9	-26.0	-18.7	-13.5	-9.6	-8.8	-8.2
E_{max}	2.8	5.0	5.0	4.8	9.3	11.0	9.9	3.0	3.5	3.5	3.5	8.3	10.9	9.7
r	-	0.929	0.932	0.871	0.622	0.303	0.138	-	0.938	0.937	0.844	0.552	0.234	0.058
RMSE	-	1.86	1.75	2.42	3.88	5.09	5.58	-	2.23	2.14	3.52	5.19	6.36	6.89
**HBDH: Q_{RK} = -1 (T)**														
Σ_{avg} E	-165.9	-138.5	-125.9	-91.0	-47.6	-15.5	-5.4	-179.6	-147.1	-124.2	-79.9	-33.2	-0.7	10.0
E_{min}	-37.7	-36.4	-36.4	-36.4	-36.4	-36.4	-36.4	-38.8	-37.8	-37.8	-37.8	-37.8	-37.8	-37.8
E_{max}	4.9	5.7	5.7	8.6	18.4	20.5	18.0	4.4	4.3	4.6	7.9	18.3	22.6	21.9
r	-	0.982	0.979	0.903	0.711	0.538	0.473	-	0.974	0.942	0.754	0.500	0.348	0.300
RMSE	-	2.18	2.60	5.18	8.67	11.27	12.17	-	2.68	4.16	7.93	11.63	13.95	14.64
**HBDH: Q_{RK} = -1 (F)**														
Σ_{avg} E	-284.8	-261.9	-246.2	-202.8	-147.1	-103.2	-83.4	-301.2	-275.9	-249.1	-195.3	-135.4	-90.3	-68.7
E_{min}	-122.4	-130.1	-126.6	-116.5	-102.3	-89.9	-80.0	-123.8	-135.3	-130.8	-121.6	-108.0	-94.1	-81.2
E_{max}	4.9	5.7	5.7	8.6	18.4	20.5	18.0	4.4	4.3	4.6	7.9	18.3	22.6	21.9
r	-	0.996	0.996	0.984	0.947	0.896	0.862	-	0.995	0.989	0.959	0.905	0.850	0.815
RMSE	-	2.41	2.65	5.33	9.35	12.64	14.36	-	3.09	4.20	7.94	12.07	15.06	16.63
**HBDH: Q_{RK} = -3 (T)**														
Σ_{avg} E	-291.0	-233.3	-179.6	-44.7	104.8	207.7	235.6	-324.4	-264.1	-206.7	-74.5	75.8	183.8	224.0
E_{min}	-31.3	-36.4	-28.2	-31.1	-30.0	-23.6	-14.3	-53.8	-65.1	-65.1	-65.1	-65.1	-65.1	-65.1
E_{max}	7.1	7.4	7.4	29.6	66.2	81.5	79.1	5.7	5.4	5.4	26.4	62.5	80.3	81.5
r	-	0.889	0.902	0.328	-0.236	-0.451	-0.559	-	0.814	0.746	0.232	-0.287	-0.450	-0.530
RMSE	-	4.93	5.52	12.75	21.42	27.00	28.44	-	6.98	7.85	13.52	21.22	26.81	28.82
**HBDH: Q_{RK} = -3 (F)**														
Σ_{avg} E	-811.6	-787.3	-724.1	-563.2	-378.5	-240.2	-181.1	-883.4	-819.6	-751.2	-590.4	-401.3	-254.2	-178.8
E_{min}	-189.2	-191.2	-185.8	-172.4	-156.3	-140.9	-127.8	-189.1	-195.9	-190.0	-179.3	-161.0	-143.5	-130.3
E_{max}	20.5	7.4	7.4	29.6	66.2	81.5	79.1	5.7	5.4	5.4	26.4	62.5	80.3	81.5
r	-	0.974	0.973	0.950	0.893	0.836	0.805	-	0.989	0.988	0.969	0.920	0.865	0.831
RMSE	-	10.22	10.49	15.49	23.35	29.21	31.63	-	7.10	7.83	13.53	21.63	27.93	31.08

Table S2. List of individual interactions between reaction kernel and surrounding moieties, depicting impact of charge damping (r_0 = 3.0 Å) onto the accuracy of the predicted interaction energies. In red, the most relevant examples are indicated, testifying to relevance of the charge attenuation. All energy values are given in kcal/mol.

MAO-A				PriA				HisA			
**Residue**	**QM**	**CH**	**CH-d**	**Residue**	**QM**	**CH**	**CH-d**	**Residue**	**QM**	**CH**	**CH-d**
Tyr58	-9.41	-10.02	-7.81	Arg142	-7.02	-4.34	-4.23	H_2O 369	-5.26	-8.94	-4.45
Tyr433	-9.16	-9.53	-9.28	Ser199	-5.30	-7.76	-3.88	H_2O 2378	-5.24	-6.02	-2.52
Asn170	-7.01	-8.07	-5.22	H_2O 1210	-4.45	-6.12	-4.14	H_2O 2011	-5.21	-7.55	-3.15
Tyr396	-6.82	-0.81	-0.82	Lys226	-3.94	-4.47	-4.30	H_2O 1569	-4.34	-6.50	-2.10
Met434	-6.48	-10.45	-4.44	Thr169	-2.06	-0.64	-0.60	Trp145	-3.09	-1.29	-1.29
H_2O 6963	-4.87	-3.79	-3.59	Ile223	-1.77	0.36	0.39	H_2O 2815	-2.58	-2.20	-2.01
H_2O 6936	-4.78	-11.77	-4.10	Asp170	-1.24	-0.62	-0.58	Hie47	-2.41	-2.54	-1.79
H_2O 9827	-4.69	-4.05	-1.71	Gly200	-0.60	-0.21	-0.20	Ala5	-0.89	0.16	0.16
H_2O 8163	-3.76	-7.00	-4.17	Ile171	-0.30	0.03	0.04	Ser202	-0.88	-0.32	-0.38
Ala57	-3.20	-1.88	-1.87	Ala8	-0.13	0.77	0.71	H_2O 1220	-0.66	-1.88	-1.72
H_2O 8090	-2.64	-11.90	-3.20	H_2O 693	-0.04	-11.00	-1.65	Ile173	-0.62	-0.17	-0.16
Lyn294	-1.95	-1.33	-1.31	H_2O 1227	0.06	0.50	0.51	Thr171	-0.49	0.31	0.30
Gln204	-1.95	1.42	1.43	Val51	0.06	0.35	0.33	Val49	-0.18	0.24	0.21
H_2O 8675	-1.67	-1.11	-1.00	H_2O 1832	0.43	0.99	1.01	Leu228	0.02	0.03	0.02
Trp386	-1.51	-0.80	-0.83	Lys173	0.66	-0.53	-0.52	Gly203	0.07	0.31	0.30
Val54	-1.36	-1.04	-1.00	H_2O 1628	1.12	1.29	1.22	Arg175	0.81	0.16	0.15
Ile169	-0.95	-0.15	-0.14	Hip49	4.02	3.98	3.62	Gly177	1.41	0.08	0.02
Gly55	-0.69	0.07	0.06					H_2O 2371	1.45	1.77	1.52
Phe341	-0.50	0.35	0.36					H_2O 1807	1.78	2.28	2.11
Phe197	-0.40	0.30	0.19					H_2O 1127	2.40	2.82	2.79
Ile196	0.30	1.64	1.67								
H_2O 7530	0.96	1.65	1.50								
H_2O 9237	2.03	2.65	2.67								
Arg40	2.94	3.40	3.38								
